# Clinical efficacy of ^99m^Tc-MIBI SPECT/CT compared with CBCT in lung biopsies: a retrospective cohort study

**DOI:** 10.3389/fonc.2026.1758732

**Published:** 2026-03-12

**Authors:** Kai Yuan, Li Long, Guangqiang Yang, Haiying Wang, Shi Yang, Yang Jiang, Tiangang Hu, Yakun Wu, Wei Qin

**Affiliations:** 1 Interventional Radiology Center, Suining Central Hospital, Suining, Sichuan, China; 2 Radiation Interventional Diagnosis and Treatment Center, Luzhou Longmatan Traditional Chinese Medicine Hospital, Luzhou, Sichuan, China; 3 Department of Interventional Medicine, Anju District People's Hospital of Suining, Suining, Sichuan, China; 4 Interventional Radiology Center and Department of Nuclear Medicine, Suining Central Hospital, Suining, Sichuan, China

**Keywords:** ^99m^Tc-MIBI SPECT/CT imaging, complications, false-negative rate, malignant lesions, percutaneous lung aspiration biopsy, positive rate

## Abstract

**Background:**

This study aimed to evaluate the clinical value of ^99m^Tc-MIBI SPECT/CT (Single-Photon Emission Computed Tomography/Computed Tomography) imaging-guided percutaneous lung aspiration biopsy by comparing its diagnostic accuracy and complication rates to those of CBCT-guided biopsy.

**Methods:**

A total of 115 patients who underwent percutaneous lung aspiration biopsy at Suining Central Hospital from September 2019 to December 2020 were included. Patients were assigned to either the ^99m^Tc-MIBI SPECT/CT-guided group (n = 34) or the CBCT-guided group (n = 81). Baseline characteristics, including age, sex, lesion location, type, and size, were statistically analyzed to ensure comparability. Bayesian multilevel logistic regression was utilized for subgroup analyses, and Clopper-Pearson exact intervals were calculated for accuracy metrics. Diagnostic accuracy and post-procedure complications were evaluated. In the SPECT/CT group, the target-to-background Ratio (TBR) uptake ratio was measured, and its predictive value for malignancy was assessed via ROC curve analysis. Interobserver reliability for TBR ratios was determined using the intraclass correlation coefficient (ICC).

**Results:**

Baseline characteristics were comparable between groups (all p > 0.05). The SPECT/CT-guided group achieved 100% diagnostic accuracy, outperforming the CBCT-guided group (93.83%). Higher TBR ratios on SPECT/CT were strongly predictive of malignancy (AUC = 1.0, ICC = 0.938). For mass-type lesions 3–3.99 cm in diameter, SPECT/CT guidance yielded significantly higher accuracy than CBCT (p = 0.027, Posterior Probability of Superiority >96%). No significant accuracy differences were observed in other lesion locations, except for superior results in the left lower lobe with SPECT/CT. Complication rates were similar (23.53% vs. 16.05%, p > 0.05).

**Conclusion:**

^99m^Tc-MIBI SPECT/CT-guided biopsy serves as a promising exploratory tool for metabolic navigation in lung biopsies. It may offer incremental value in identifying viable tumor tissue within intermediate-sized masses without compromising safety. Given the pilot nature of this study, these findings are hypothesis-generating and necessitate confirmation through prospective, multicenter randomized trials.

## 1 Introduction

Malignant tumors pose a significant threat to human health. According to the 2022 global cancer statistics, lung cancer remains the most prevalent type of cancer and the leading cause of cancer-related deaths in China (1). Accurate pathological typing and clinical staging are critical in the diagnosis and treatment of lung cancer, directly impacting patient survival rates (2, 3). Therefore, precise puncture biopsy is crucial (4).

Early tissue biopsy is vital for characterizing lung lesions (5). When imaging detects occupational lung lesions, confirming whether they are primary or metastatic is necessary for treatment planning. For patients with poor response to radiotherapy or chemotherapy, percutaneous lung aspiration biopsy provides genetic information to guide targeted or immunotherapy. Imaging-guided percutaneous lung aspiration biopsy is preferred in clinical practice due to its minimally invasive nature, efficiency, and low complication rates (6). However, its accuracy can be limited by false negatives, especially in complex cases such as mass-type lesions with compression or necrosis, and diffuse small nodules (7), highlighting the need for improved imaging guidance.

In clinical practice, Cone Beam Computed Tomography (CBCT) and Single-Photon Emission Computed Tomography combined with Computed Tomography (SPECT/CT) play pivotal roles in biopsy-guided diagnosis of lung tumors. CBCT is widely used for its high-resolution imaging and real-time guidance but may be less effective in complex cases with intricate anatomy or indistinct lesion borders. SPECT/CT, especially with the radiotracer ^99m^Tc-methoxyisobutylisonitrile (^99m^Tc-MIBI), provides enhanced metabolic imaging, aiding the differentiation between malignant and benign lesions (8), a particular advantage in early-stage tumors or when tumor type differentiation is needed.

Research on SPECT/CT in lung tumor evaluation has increased in recent years (9). Studies demonstrated high sensitivity and specificity of SPECT/CT with ^99m^Tc-MIBI for lung cancer diagnosis. Nosotti et al. (10), reported a sensitivity of 85.7% and specificity of 100%, while Nikoletic et al. (11), found sensitivity and specificity of 89.8% and 100%, respectively, in 116 patients with suspected lung cancer. The mechanism is thought to involve the negative potential difference between the cell and mitochondrial membranes (12, 13). Additionally, combining SPECT/CT functional imaging with percutaneous lung aspiration biopsy has shown promising preliminary results (14). Qian et al. (15) further confirmed the clinical feasibility and safety of SPECT/CT-guided percutaneous biopsy.

Despite the potential advantages of ^99m^Tc-MIBI SPECT/CT, comprehensive clinical data directly comparing its effectiveness with CBCT in guiding lung biopsies are lacking. This study addresses this gap by evaluating the clinical utility of ^99m^Tc-MIBI SPECT/CT-guided percutaneous lung aspiration biopsy, with a focus on accuracy and postoperative complication rates compared to CBCT-guided procedures. By analyzing 115 cases, we assessed the practical benefits and limitations of this approach, aiming to enhance lung cancer diagnostics and patient outcomes.

## 2 Materials and methods

### 2.1 Participants

**Figure 1:**
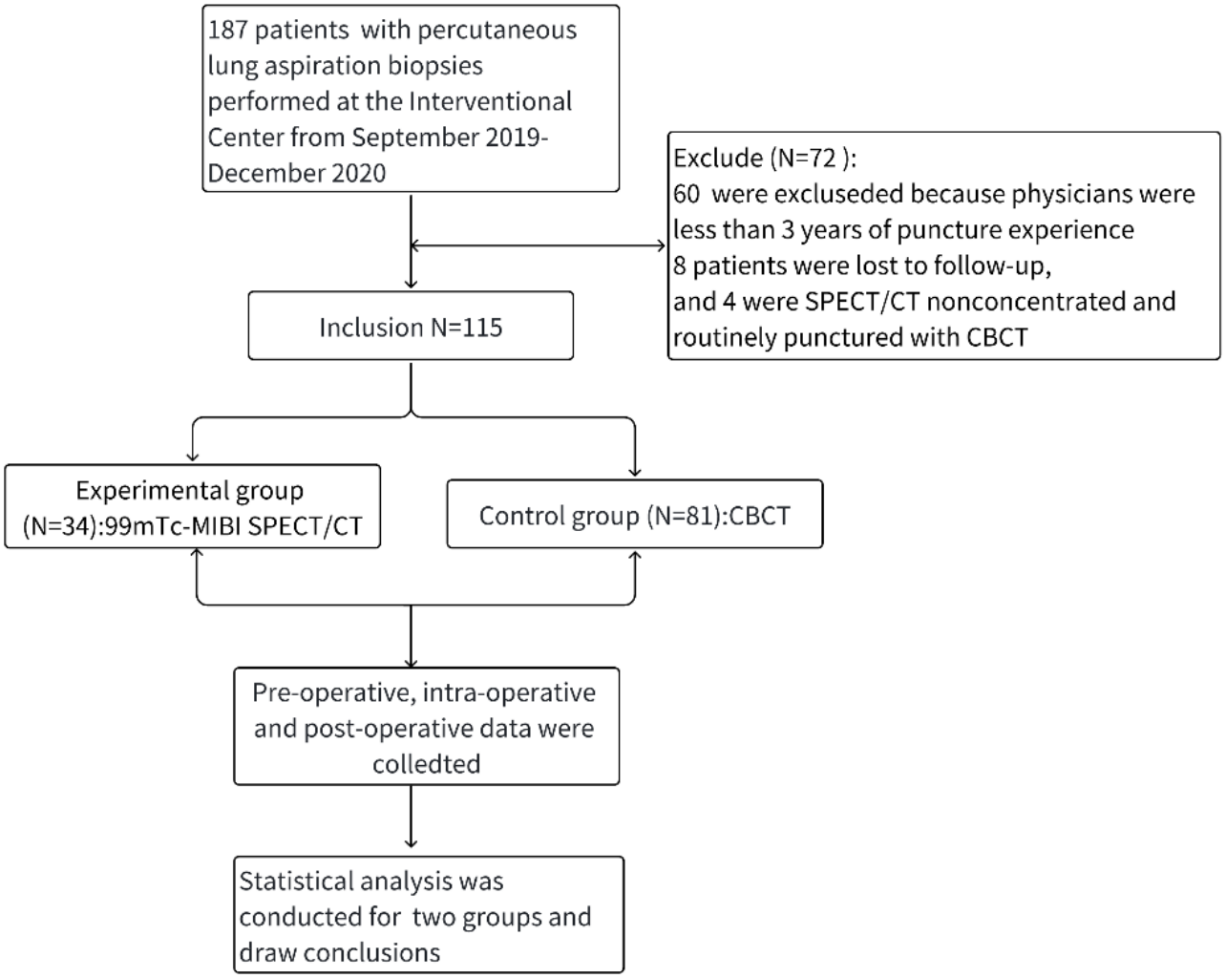
Research design diagram.

This retrospective study was approved by the Suining Central Hospital Ethics Committee. Informed consent for the use of clinical data was obtained from all patients or their guardians. We initially screened 187 patients who underwent percutaneous lung aspiration biopsy between September 2019 and December 2020 (**Figure 1**).

Inclusion criteria were: (1) patients who underwent CBCT- or ^99m^Tc-MIBI SPECT/CT-guided percutaneous lung aspiration biopsy; (2) procedures performed by interventionalists with more than three years of experience; and (3) guidance method selected according to lesion characteristics and equipment availability. Exclusion criteria were: (1) use of other guidance techniques; (2) operators with less experience; (3) significant comorbidities or lung conditions increasing procedural risk or confounding efficacy; (4) recent thoracic interventions; and (5) absence of lesion uptake on SPECT/CT, precluding its use.

A total of 115 patients met the inclusion criteria, with 34 in the experimental (SPECT/CT) group and 81 in the control (CBCT) group. The smaller sample size in the SPECT/CT group reflects the limited availability and selective, non-routine use of this technology during the study period. Logistical factors, such as equipment access and the Nuclear Medicine Department being in a separate hospital district, further restricted case numbers. This imbalance may affect the statistical power of group comparisons.

### 2.2 Instruments and materials

This study employed specialized instruments and materials to ensure the precision and safety of percutaneous lung aspiration biopsy procedures. The primary imaging modality was the Artis zee ceiling system (Siemens, Germany), set at 125 kV and 1000 mA, chosen for its high-resolution, real-time imaging capabilities essential for accurate biopsy guidance.

Biopsies were performed using 18G semi-automatic cutting biopsy needles (Argon Medical Devices), selected for their reliability and efficiency in obtaining high-quality lung tissue samples while minimizing patient discomfort and complication rates.

Each procedural kit included a 5 ml syringe, 5 ml of 2% lidocaine hydrochloride for local anesthesia, povidone-iodine skin disinfectant for pre-procedure skin preparation, a tissue fixation solution for pathological preservation, 100 ml of 0.9% saline, surgical sterile gloves, two pieces of gauze, and adhesive tape for post-procedure management.

For SPECT/CT-guided procedures, the diagnostic agent ^99m^Tc-MIBI (Atomic High Tech Co., Ltd.) was used, with a radiochemical purity exceeding 95%, ensuring optimal imaging contrast and precise localization of target biopsy sites.

### 2.3 Operation procedures

#### 2.3.1 Preoperative preparation

1. Operators were thoroughly trained and demonstrated proficiency in the indications and contraindications for percutaneous lung aspiration biopsy. Prior to biopsy, all subjects underwent comprehensive preoperative evaluations, including complete blood count, coagulation profile, liver and kidney function tests, blood biochemistry, electrocardiogram, and chest CT (with contrast, as needed) to assess vascular flow at the puncture site and to rule out contraindications for biopsy of lung lesions.
2. Patients and/or their families were informed about the purpose, procedure, and potential complications of the biopsy, and written informed consent was obtained.
3. Preoperative respiratory training was provided to patients to promote cooperation during the procedure, facilitating smooth sampling and minimizing complications.

#### 2.3.2 CBCT-guided percutaneous lung aspiration biopsy procedure

1. The biopsy target was identified using a cognitive targeting approach based on pre-procedural contrast-enhanced CT images. The puncture site was marked using the CBCT laser positioning system and a metal grid, with a safe path planned based on enhanced CT images. After standard skin disinfection and sterile draping, local anesthesia was administered with lidocaine. An 18G semi-automatic cutting biopsy needle (Argon Medical Devices) was used for tissue sampling.

Patients maintained calm breathing and a steady position during the procedure. Under CBCT guidance and fluoroscopy, the needle was advanced to the target area, avoiding critical structures. The patient was instructed to hold their breath during pleural puncture and tissue sampling to minimize complications. Tissue integrity was confirmed, and samples were placed in fixative for pathology. The process was repeated as needed until an adequate specimen was obtained. The puncture site was dressed, and samples were sent to the Department of Pathology for examination.

2. After the procedure, CBCT was used to check for complications. Patients rested supine for at least 4 hours and were monitored for adverse events, which were assessed by chest radiograph or CT when necessary.

#### 2.3.3 ^99m^Tc-MIBI SPECT/CT imaging CBCT-guided percutaneous lung puncture biopsy procedure

1. Patients received an intravenous injection of 20 mCi ^99m^Tc-MIBI, followed by 120 minutes of rest with oxygen. Delayed phase SPECT/CT scans were acquired and fused using Siemens Workstation MI. Two blinded senior nuclear medicine physicians reviewed the fused images to identify the area of highest ^99m^Tc-MIBI uptake.
2. The biopsy target and optimal puncture path were determined based on the region of maximal tracer uptake. If uptake was minimal or absent, standard CBCT-guided biopsy methods were used.
3. The biopsy procedure and post-procedure management followed the protocols described in section 2.3.2, with adjustments based on SPECT/CT findings. Needle trajectory planning incorporated both metabolic and anatomical imaging for precise targeting and safety.

For each lesion, two nuclear medicine physicians independently placed a region of interest (ROI) over the area of maximum uptake and a background ROI on normal lung. The lesion-to-background (TBR) uptake ratio was calculated, and interobserver agreement was evaluated using the intraclass correlation coefficient (ICC).

### 2.4 Image interpretation and localization

To ensure the objectivity of the target localization, all ^99m^Tc-MIBI SPECT/CT images were retrospectively reviewed by two senior nuclear medicine physicians. The reviewers were blinded to the final histopathological results and other clinical outcomes to prevent expectation bias during the identification of the puncture site.

A standardized qualitative consensus protocol was followed: the two physicians independently identified the intra-lesional site with the highest tracer accumulation. Any discrepancies regarding the optimal localization for biopsy guidance were resolved through mutual discussion until a consensus was reached. This independent review process ensured that the identified puncture sites were determined solely based on the functional imaging characteristics of the ^99m^Tc-MIBI SPECT/CT scans.

### 2.5 Pathologic diagnostic criteria

A biopsy was considered positive if histopathology confirmed malignancy, supported by at least one gold standard (16): malignant pathology after surgical resection, consistent metastatic foci in other organs, or clear clinical evidence such as significant lesion regression after anti-tumor therapy.

A negative biopsy was defined as histopathology indicating a benign lesion, validated by one of the following: benign surgical pathology, marked lesion reduction or disappearance after anti-inflammatory or anti-tuberculosis therapy, or stable/reduced lesion size and morphology on serial CT scans at 3, 6, and 12 months without progression. Although 12-month stability does not entirely rule out indolent malignancy, it is an accepted clinical threshold for benignity in the absence of suspicious findings.

For patients with negative biopsy results who proceeded to surgery due to suspected false negatives, surgical intervention was considered based on comprehensive clinical assessment. Indications included lesion progression or suspicious imaging features, persistent or worsening symptoms unexplained by benign disease, or a high-risk clinical background coupled with concerning radiological findings. All surgical decisions were thoroughly documented in the medical records.

A false-negative result was defined as a biopsy initially interpreted as benign, with subsequent evidence confirming malignancy. This included cases where repeat biopsy or surgical resection established malignancy, or where lesion progression or new metastatic lesions appeared during follow-up and were confirmed as malignant by further pathological examination.

### 2.6 Statistical analysis

Patient data (age, gender, lesion characteristics, biopsy histopathology, and puncture-related complications) were collected systematically. Analyses were performed using SPSS 23.0 and STATA 17.0. Normally distributed data were assessed with independent t-tests (mean ± SD), and non-normal data with the Mann-Whitney U test. Categorical variables were compared using Chi-square or Fisher’s exact tests; p < 0.05 was considered significant. A sensitivity power analysis was performed to determine the detectable effect size given the final sample size (N = 115; experimental group n = 34, control group n = 81) with a two-sided alpha of 0.05 and 80% power.

Diagnostic accuracy, sensitivity, and specificity for ^99m^Tc-MIBI SPECT/CT and CBCT-guided biopsies were calculated by comparing biopsy results with final histopathological diagnoses. The TBR ratio’s predictive value for biopsy positivity was assessed using the receiver operating characteristic (ROC) curve analysis, with AUC reported. Cases were grouped by median TBR ratio to compare diagnostic yields. Interobserver agreement for TBR ratio was evaluated with the intraclass correlation coefficient (ICC), and Cohen’s kappa was used to assess agreement for visual classification of MIBI uptake (high vs. low).

To ensure the robustness of subgroup findings and address the inherent limitations of small sample sizes and potential statistical separation, Bayesian multilevel logistic regression was employed for all stratified analyses, including lesion type, diameter, and puncture location. This hierarchical approach utilized a partial-pooling strategy, allowing for information sharing across strata to stabilize estimates for small subgroups and mitigate the risk of overfitting. Model parameters, including posterior means, 95% credible intervals (CrIs), and the posterior probability of superiority (PPoS), were estimated via Markov Chain Monte Carlo (MCMC) methods. A PPoS > 95% was considered to provide strong evidence of the experimental intervention’s advantage within a specific stratum.

## 3 Results

### 3.1 Comparison of general demographic data between two groups

The experimental group (n = 34) included patients aged 42–80 years (mean 64.9 ± 8.9), with 25 males and 9 females, and lesion diameters ranging from 1.5 to 9.8 cm (mean 3.82 ± 1.96). The control group (n = 81) comprised patients aged 41–86 years (mean 62.7 ± 9.9), with 36 males and 45 females, and lesion diameters from 1.2 to 10.3 cm (mean 3.33 ± 1.45).

**Table 1** summarizes the demographic and baseline lesion characteristics, including age, gender, lesion location, type, and diameter. Statistical analysis was performed using the t-test for age, Mann-Whitney U test for lesion diameter, and chi-square or Fisher’s exact tests for categorical variables. No statistically significant differences were found between the two groups for any of the variables (all p > 0.05), indicating demographic comparability between groups. This similarity strengthens the internal validity of the study, ensuring that outcome differences are likely attributable to the intervention rather than baseline disparities.

**Table 1:** Patient demographic data between two groups.

Variables	Experimental group(n = 34)	Control group (n = 81)	*t/Z/χ^2^*	*p*
Average age (Mean ± SD)	64.88 **±** 8.89	62.67 **±** 9.89	1.129	0.261^a^
Gender(n, %)				
Men	25(73.53)	36(44.44)	3.248	0.072^b^
Women	9(26.47)	45(55.56)		
Percutaneous lesion location (n, %)			8.970	0.095^c^
Right upper lobe	12(35.29)	26(32.10)		
Right middle lobe	3(8.82)	10(22.22)		
Right lower lobe	4(11.77)	18(12.35)		
Left upper lobe	4(11.77)	18(22.22)		
Left lower lobe	9(26.47)	8(9.88)		
Hilum	2(5.88)	1(1.23)		
Percutaneous lesion types (n, %)			2.844	0.397^c^
Solitary nodule type	4(11.76)	16(19.75)		
Multinodular nodule type	8 (23.53)	26(32.10)		
Cavitary type	1(2.94)	3(3.70)		
Mass type	21 (61.76)	36(44.44)		
Average diameter of percutaneous lesions (cm)	3.82 **±** 1.96	3.33 **±** 1.45	−0.902	0.367^d^

^a^indicates the use of the t-test.

^b^indicates the use of the chi-square test.

^c^indicates the use of Fisher’s exact test for testing.

^d^means the use of Mann-Whitney U test.

### 3.2 Accuracy rates of puncture results in two groups

**Figure 2:**
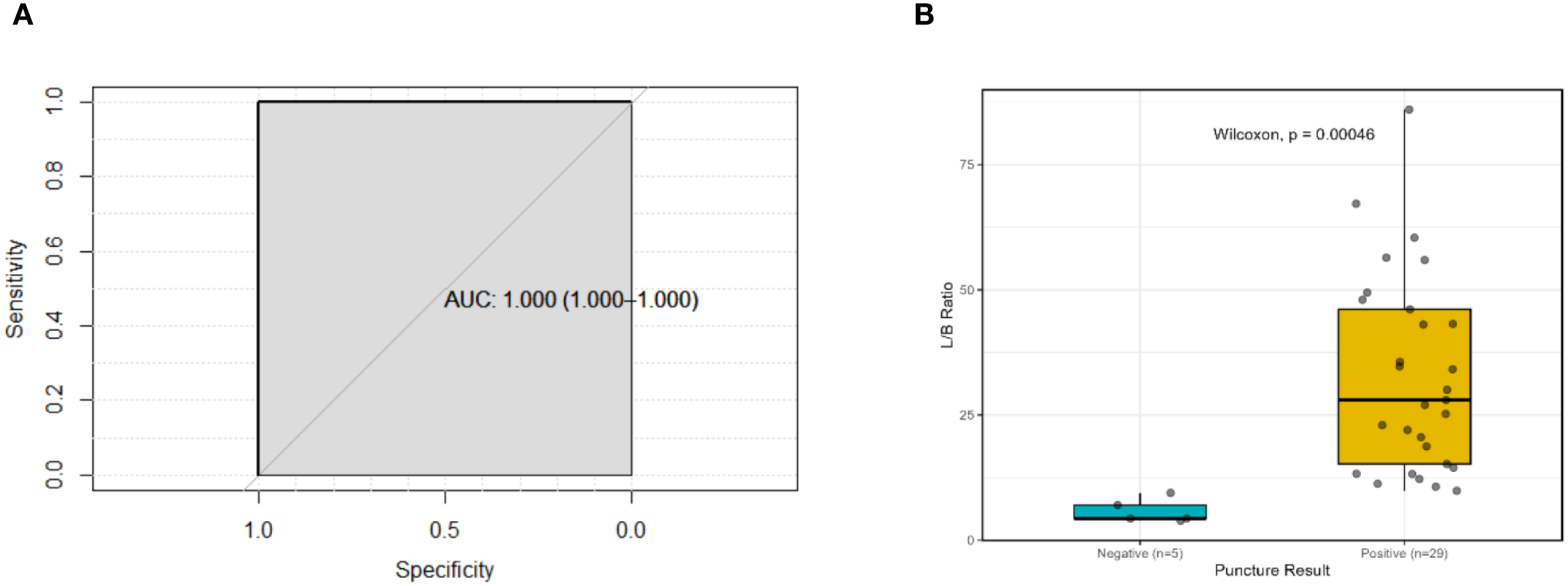
Quantitative analysis of ^99m^Tc−MIBI uptake (TBR) as a predictor of pulmonary malignancy: **(A)** Stratified bootstrap-validated ROC analysis; **(B)** Distribution and separation analysis of TBR ratios.

In the experimental group, quantitative analysis of ^99m^Tc-MIBI SPECT/CT images yielded a median TBR uptake ratio of 24.095 (range: 3.88–86.02; IQR: 11.98–43.94). The ROC analysis demonstrated that the TBR ratio achieved an Area Under the Curve (AUC) of 1.00. To assess the robustness of this finding and account for the potential overfitting due to the small sample size, a stratified bootstrap validation (2,000 iterations) was performed (**Figure 2A**). The resulting 95% confidence interval (CI) remained 1.00–1.00, indicating a stable and complete separation between the two groups within the current cohort. Specifically, the negative group (range: 3.88–9.43) and positive group (range: 9.85–86.02) exhibited no overlapping values (**Figure 2B**). No overlap in TBR values was observed between the pathologically confirmed negative and positive cases.

The experimental group achieved 100% sensitivity, specificity, and accuracy, correctly identifying all 34 lesions (**Table 2**). The 95% CI for accuracy in the experimental group was 89.7%–100.0% (Clopper-Pearson exact method). In the control group, diagnostic accuracy was 93.83%, with a 95% CI of 86.2%–98.0% (Clopper-Pearson). Sensitivity and specificity for the control group were 90.91% and 100%, respectively. Five false negatives (6.17%) were observed in the control group, whereas no false results occurred in the experimental group.

**Table 2:** Accuracy comparison of puncture results between two groups.

Results	Experimental group (*n*, %)	Control group (*n*, %)
Negative	5(14.71)	26(32.10)
Positive	29(85.29)	50(61.73)
False negative	0(0.00)	5(6.17)
False positive	0(0.00)	0(0.00)
Accuracy	100%	93.83%
Sensitivity	100%	90.91%
Specificity	100%	100%

None of the 31 patients with negative biopsy results underwent surgical resection due to concerns about possible false negatives. All negative cases were followed up according to standard clinical protocols.

To further validate these findings, bootstrap resampling (1,000 iterations) was performed. The bootstrap-estimated accuracy for the experimental group remained 100% (95% CI: 100.0%–100.0%), while the control group showed a bootstrap accuracy of 93.8% (95% CI: 88.9%–98.8%). Although Chi-square analysis indicated that the difference in biopsy outcomes between the two groups did not reach formal statistical significance (χ^2^ = 3.679, p = 0.067), the overlapping yet distinct confidence intervals suggest a trend toward improved diagnostic reliability with ^99m^Tc-MIBI SPECT/CT guidance.

### 3.3 Accuracy comparison across lesion types

Puncture accuracy was evaluated across various lesion types, assessing whether imaging modality influences the precision of percutaneous lung punctures for different lesions (**Table 3**). Univariate analysis identified mass-type lesions (diameter > 3 cm) as the primary subgroup benefiting from the experimental intervention (p = 0.03). Stratified analysis of these masses (**Table 4**) revealed that the diagnostic advantage was most pronounced in the 3.0–3.99 cm diameter range (p = 0.027), suggesting a potential performance gain for the experimental approach within this size range; however, these subgroup findings should be considered exploratory.

**Table 3:** Comparison of the accuracy of puncture results between the two groups for different lesion types.

Lesion types	Results	Experimental group (*n*, %)	Control group (*n*, %)	χ^2^	*p*
Solitary type	Negative	1(25.00)	3(18.75)	–	1.000^a^
	Positive	3(75.00)	13(81.25)		
	False negative	0(0.00)	0(0.00)		
Multinodular type	Negative	2 (25.00)	11(42.31)	1.785	0.393^a^
	Positive	6(75.00)	12(46.15)		
	False negative	0(0.00)	3(11.54)		
Cavitary type	Negative	1(100)	2(66.67)	–	1.000^a^
	Positive	0(0.00)	1(33.33)		
	False negative	0(0.00)	0(0.00)		
Mass type	Negative	1(4.76)	10(27.78)	5.811	0.030^a,*^
	Positive	20(95.24)	24(66.67)		
	False negative	0(0.00)	2(5.55)		

^a^denotes test by Fisher’s exact method. *means that for the mass type, the comparison of the accuracy of the puncture results between two groups showed a statistically significant difference of *p<0.05*.

**Table 4:** Comparison of accuracy rates in percutaneous procedures for mass-type lesions of different diameters between two groups.

Diameter(cm)	Results	Experimental group (*n*, %)	Control group (*n*, %)	χ^2^	*p*
3–3.99	Negative	0(0.00)	5(33.33)	5.923	0.027^a^*
	Positive	12(100.00)	9(60.00)		
	False negative	0(0.00)	1(6.67)		
4–4.99	Negative	2(40.00)	5(29.41)	–	1.000^a^
	Positive	3(60.00)	12(70.59)		
	False negative	0(0.00)	0 (0.00)		
≥ 5	Negative	0(0.00)	3(37.5)	–	0.209^a^
	Positive	6(100.00)	5(62.5)		
	False negative	0(0.00)	0(0.00)		

^a^indicates that the test was performed using Fisher’s exact test. The asterisk (*) denotes statistical significance with a p-value < 0.05.

To address potential heterogeneity across lesion types and the inherent limitations of small subgroup sizes, a Bayesian multilevel logistic regression was further employed. The models utilized a partial-pooling approach. This facilitated information sharing across strata, thereby stabilizing the estimates for small subgroups and providing a more robust assessment of the overall intervention effect despite the limited sample size. The lesion-type model yielded a posterior mean log-odds coefficient of 2.50 (95% CrI: –0.20 to 6.00; OR ≈ 12.18, 95% CrI: 0.82–403.43), while the diameter-stratified model showed a mean coefficient of 2.48 (95% CrI: –0.20 to 6.03; OR ≈ 11.94, 95% CrI: 0.82–415.72). Despite wide CrIs due to limited sample sizes in specific strata, the posterior probability of superiority (PPoS) exceeded 96% in both analyses, providing strong evidence favoring the experimental intervention. These findings indicate a robust clinical trend of substantial performance gains with the experimental intervention, notably within the 3.0–3.99 cm mass category where the marginal benefit of SPECT/CT is most clinically relevant.

Representative cases for each lesion type are illustrated in **Supplementary Figures S1**-**S5**, providing visual context for the textural and morphological diversity observed in the study group.

### 3.4 Accuracy analysis based on puncture locations

Puncture accuracy rates were compared between the experimental and control groups across different lung regions to assess whether imaging modality influenced diagnostic performance by location. Initial subgroup analysis (**Table 5**) indicated superior accuracy in the experimental group for lesions in the left lower lobe (p = 0.029), while no significant differences were observed at other puncture sites.

**Table 5:** Comparison of puncture result accuracy across two groups for lesions located in different anatomical locations.

Puncture lesion locations	Result	Experimental group (*n*, %)	Control group (*n*, %)	χ^2^	*p*
Right upper lobe	Negative	5(41.67)	10(38.46)	0.557	1.000^a^
	Positive	7(58.33)	15(57.69)		
	False negative	0(0.00)	1(3.85)		
Right middle lobe	Negative	0(0.00)	3(30.00)	–	1.000^a^
	Positive	3(100)	5(50.00)		
	False negative	0(0.00)	2(20.00)		
Right lower lobe	Negative	0(0.00)	2(11.11)	0.489	0.484^a^
	Positive	4(100)	16(88.89)		
	False negative	0(0.00)	0(0.00)		
Left upper lobe	Negative	0(0.00)	8(44.44)	3.233	0.289^a^
	Positive	4(100)	9(50.00)		
	False negative	0(0.00)	1(5.56)		
Left lower lobe	Negative	0(0.00)	3(37.50)	5.326	0.029*
	Positive	9(100)	4(50.00)		
	False negative	0(0.00)	1(12.50)		
Hilum	Negative	0(0.00)	0(0.00)	–	–
	Positive	2(100)	1(100)		
	False negative	0(0.00)	0(0.00)		

^a^indicates that the test was performed using *Fisher’s* exact test. The asterisk (*) denotes statistical significance with a p-value < 0.05.

To address the inherent limitations of small subgroup sizes (e.g., n = 3 at specific sites) and potential site-specific heterogeneity, a Bayesian multilevel logistic regression was employed with lesion site as a random intercept. After adjusting for age and gender, the posterior mean for the group coefficient was 2.56 (OR ≈ 12.9, 95% CrI: –0.12 to 6.10). Although the 95% CrI marginally crossed the null, the model yielded a 96.76% posterior probability that ^99m^Tc-MIBI SPECT/CT guidance was associated with superior diagnostic accuracy compared to the control modality. The estimated variance for the random intercept (site) was 1.42 (95% CrI: 0.25 to 5.95), justifying the inclusion of inter-site variability. These findings suggest a strong clinical signal favoring the experimental group, indicating that the observed diagnostic advantages remain consistent across anatomical locations when adjusting for hierarchical data structures.

### 3.5 Complications of puncture

Complications occurred in 21 patients: 19 developed pneumothorax (7 in the experimental group, 12 in the control group), and 2 experienced hemoptysis (one in each group). The overall complication rates were 23.53% in the experimental group and 16.05% in the control group, with no statistically significant difference (p *=* 0.397), aligning with previous reports (17). Detailed complication rates are presented in **Table 6**.

**Table 6:** Comparison of the incidence of post-puncture complications between the two groups.

Complications	Experimental group (*n*, %)	Control group (*n*, %)	χ^2^	*p*
None	26(76.47)	68(83.95)	1.521	0.397^a^
Pneumothorax	7(20.59)	12(14.81)		
Hemoptysis	1(2.94)	1(1.24)		

^a^indicates that *Fisher’s* exact test was used.

To account for potential confounding factors, a multivariable logistic regression analysis was performed, adjusting for age, gender, lesion site, and lesion type. After adjustment, the experimental group was not associated with a significantly increased risk of complications compared with the control group (adjusted OR = 1.252, 95% CI: 0.405–3.872, p = 0.697). These findings suggest that the experimental approach does not increase procedural risk, even when considering individual patient and lesion characteristics.

### 3.6 Sensitivity analysis of surgically confirmed cases

To assess the robustness of the diagnostic criteria, a sensitivity analysis was performed on a subset of 11 patients (4 in the experimental group, 7 in the control group) who underwent surgical resection. All 11 lesions were pathologically confirmed as malignant, representing various subtypes including adenocarcinoma, squamous cell carcinoma, and metastatic disease. In the experimental group, ^99m^Tc-MIBI SPECT/CT correctly identified all 4 malignancies (Sensitivity: 100%, 95% CI: 39.8%–100.0%), including one case of invasive mucinous adenocarcinoma. In the control group, the sensitivity was also 100% (7/7, 95% CI: 59.0%–100.0%). Restricting the analysis to this surgically confirmed subset did not alter the overall diagnostic conclusions observed in the full cohort, supporting the robustness of the primary analysis under the strictest reference standard.

## 4 Discussion

### 4.1 Comparative diagnostic performance and the value of functional imaging

This study evaluated the diagnostic accuracy of ^99m^Tc-MIBI SPECT/CT versus CBCT in guiding percutaneous lung punctures. ^99m^Tc-MIBI SPECT/CT achieved 100% accuracy with no false negatives, significantly outperforming CBCT’s 93.83% accuracy and five false negatives. The superiority of ^99m^Tc-MIBI SPECT/CT was especially evident in mass-type lesions, with statistically significant accuracy improvement for lesions 3–3.99 cm in diameter (*p* = 0.027).

In the control group, false negatives were initially misdiagnosed as benign—four as inflammation and one as necrosis—but later confirmed as two metastatic cancers (from esophageal and breast primaries), two adenocarcinomas, and one squamous cell carcinoma. In contrast, the experimental group’s five benign lesions showed no significant ^99m^Tc-MIBI uptake, some displaying the “vacuolar sign” of benignity. All 29 malignant lesions in this group showed uptake: eight uniformly, 21 non-uniformly. These results highlight ^99m^Tc-MIBI SPECT/CT’s strong potential for distinguishing malignant from benign pulmonary lesions based on uptake patterns.

Compared to other advanced guidance modalities, ^99m^Tc-MIBI SPECT/CT offers a unique balance between diagnostic yield and technical accessibility. While PET/CT fusion is often regarded as the gold standard for metabolic targeting, its high cost and the requirement for an on-site cyclotron limit its routine use in many centers (18). In contrast, SPECT/CT provides comparable functional localization at a lower cost. Furthermore, while contrast-enhanced ultrasound (CEUS) provides real-time imaging without radiation, its diagnostic reach is fundamentally constrained by the acoustic impedance mismatch between the chest wall and the aerated lung parenchyma (19). This physical limitation results in the total reflection of ultrasonic energy, rendering CEUS ineffective for lesions that are not in direct contact with the pleura. In contrast, ^99m^Tc-MIBI SPECT/CT is unaffected by intrapulmonary gas, allowing for the precise localization of deep-seated metabolic targets.

### 4.2 Subgroup insights in mass-type lesions and challenging anatomical sites

While our study found significantly improved accuracy for mass-type lesions 3–3.99 cm in diameter (*p* = 0.027) and higher puncture accuracy in the left lower lobe (*p* = 0.029), no such advantage was observed for multinodular, solitary, or cavitary lesions (11). Limited sample size per diameter category likely influenced these findings, underscoring the need for larger cohorts. The distinctive challenges of the left lower lobe, due to its anatomical complexity and proximity to the heart and diaphragm, may explain the observed differential performance (20). ^99m^Tc-MIBI SPECT/CT offers superior metabolic imaging, enhancing lesion delineation in challenging regions and improving puncture accuracy. In this study, the experimental group used SPECT/CT to target metabolically active areas, while the CBCT-guided control group prioritized safer, possibly less representative paths. Although CBCT improves safety, it may miss malignant foci. In contrast, SPECT/CT’s ability to localize hypermetabolic zones supports more targeted biopsies, increasing diagnostic yield, especially in mass-type lesions.

In distinguishing between benign and malignant lung lesions, our study capitalized on differential ^99m^Tc-MIBI uptake patterns observed in the delayed phase of imaging. As reported by Tan et al. (21), benign lesions such as tuberculosis and inflammatory pseudotumors show high initial uptake that rapidly decreases in the delayed phase, while malignant tumors maintain high uptake throughout. Accordingly, all scans were performed in the delayed phase to improve diagnostic accuracy by highlighting the sustained radiotracer retention characteristic of malignancy.

### 4.3 Biological rationale for ^99m^Tc-MIBI uptake and protocol optimization

To position the clinical utility of ^99m^Tc-MIBI, it is essential to contrast its biological rationale with that of ^18^F-fluorodeoxyglucose (^18^F-FDG), the current benchmark in metabolic imaging. While ^18^F-FDG uptake reflects glucose hypermetabolism mediated by GLUT-1 transporters (22), ^99m^Tc-MIBI is a lipophilic cation that accumulates within cells primarily in response to mitochondrial membrane potential and increased cellular density (23). This mechanistic distinction is clinically pivotal: ^18^F-FDG frequently yields false-positive results in active inflammatory or infectious processes, which are common in pulmonary pathology. In contrast, ^99m^Tc-MIBI often demonstrates higher specificity in such contexts. Furthermore, ^99m^Tc-MIBI offers significant practical advantages; the 6-hour half-life of ^99m^Tc (versus 110 minutes for ^18^F) and its derivation from widely accessible molybdenum-technetium generators make it a more cost-effective and logistically feasible option for centers lacking on-site cyclotrons (24).

Within this biological framework, individual differences in MIBI uptake, driven by each lesion’s unique metabolic traits, highlight the need for personalized diagnostics. Uptake concentration reflects internal lesion heterogeneity, making it critical to biopsy regions with highest MIBI uptake to obtain tissue truly representative of the pathology. This approach contrasts with methods relying solely on quantitative imaging metrics like SUVmax or T/N ratio, which can vary widely across patients and lesion types (25). Relying exclusively on these values, without accounting for biological context, risks false negatives due to diverse lesion responses to pro-tumorigenic tracers (26).

In our study, all patients received oxygen inhalation and rested for two hours following ^99m^Tc-MIBI injection prior to SPECT/CT imaging. This protocol was adopted in accordance with previous studies suggesting that higher arterial oxygen saturation may enhance ^99m^Tc-MIBI uptake in metabolically active tumor tissues. For example, Cermik et al. (27) and Dong et al. (28) reported improved sensitivity and specificity of ^99m^Tc-MIBI imaging with oxygen supplementation. Similarly, all patients underwent delayed-phase imaging, as prior reports indicate that malignant lesions tend to retain ^99m^Tc-MIBI uptake in delayed scans, whereas benign lesions often show reduced uptake (21). While our retrospective design precluded independent assessment of oxygen inhalation and delayed imaging, both elements were included to align with best practices and enhance diagnostic performance. Future prospective studies are warranted to clarify their individual contributions.

### 4.4 Safety and clinical implementation

The complication rates were 23.53% in the experimental group and 16.05% in the control group, with a p-value of 0.05, indicating no statistically significant difference. The main complications were pneumothorax and hemoptysis. Their occurrence was more closely associated with lesion and tissue characteristics, as well as operator skill and experience, rather than the puncture technique. These results are consistent with previous reports (29, 30), supporting the safety of ^99m^Tc-MIBI SPECT/CT-guided percutaneous lung aspiration biopsy regardless of the biopsy approach.

Although ^99m^Tc-MIBI SPECT/CT provides a metabolic guidance for lesion targeting, its role in the lung biopsy workflow must be weighed against the increased procedural complexity and resource utilization. While tracer uptake requirements extend the total hospital stay, a consistent interventional duration ensures that procedural throughput remains unaffected. From a health economics perspective, preventing even a small fraction of repeat biopsies—necessitated by the 6.17% false-negative rate in the CBCT group—offsets the incremental costs of functional imaging. This is especially critical for lesions with extensive necrosis, where SPECT/CT-derived TBR ratios offer the metabolic assessment necessary to secure a diagnostic sample during the initial procedure.

### 4.5 Limitations and directions for future research

This study has several limitations. Its retrospective, non-randomized design introduces selection bias, as ^99m^Tc-MIBI SPECT/CT was mainly used for complex or ambiguous lesions and was subject to equipment availability. Consequently, the SPECT/CT group included more complex cases and had a smaller sample size due to logistical constraints and COVID-19 restrictions, potentially limiting generalizability and statistical power. Notably, the observed 100% accuracy and the corresponding bootstrap confidence intervals should be interpreted as a preliminary proof-of-concept. Due to the limited number of negative cases (n = 5), these results likely represent an optimistic estimation and require validation in larger, prospective multicenter cohorts. While a sensitivity power analysis (N = 115) confirmed the study was powered to detect a minimum absolute difference of 22.37% in diagnostic proportions, it may lack the sensitivity to identify more subtle differences between modalities. Consequently, these findings should be interpreted as hypothesis-generating rather than definitive. Prospective, randomized studies with balanced group sizes are needed to validate these findings and reduce allocation bias. Second, ^99m^Tc-MIBI is a general tumor imaging agent rather than one specific for lung tumors. Future studies employing lung tumor-specific tracers may further enhance diagnostic specificity and sensitivity. Third, although a 12-month period of radiological stability is commonly used to classify lesions as benign, this interval may not exclude indolent or slow-growing malignancies, such as certain adenocarcinoma subtypes. Longer-term follow-up is necessary to minimize the risk of misclassification.

Despite these limitations, our findings suggest that ^99m^Tc-MIBI SPECT/CT-guided percutaneous lung biopsy may improve diagnostic accuracy in selected patients, particularly when standard imaging is inconclusive. Further multicenter, prospective studies are warranted to validate these results.

## 5 Conclusion

The exploratory study demonstrated that ^99m^Tc-MIBI SPECT/CT imaging-guided percutaneous lung aspiration biopsy has high diagnostic accuracy, particularly for mass-type lesions measuring 3.0–3.99 cm. While the experimental group achieved a point estimate of 100% accuracy, these results should be interpreted cautiously as hypothesis-generating rather than definitive, given the limited sample size and retrospective design. Importantly, multivariable-adjusted analysis confirmed that the safety profile of SPECT/CT-guided biopsy is comparable to that of conventional CBCT. These preliminary findings suggest that metabolic navigation may serve as a promising precision tool for complex or previously indeterminate pulmonary lesions, although large-scale, prospective randomized controlled trials are required to validate its incremental clinical utility and cost-effectiveness.

## Data Availability

The original contributions presented in the study are included in the article/**Supplementary Material**. Further inquiries can be directed to the corresponding authors.
