# Supplementary material for: Clinical efficacy of 99mTc-MIBI SPECT/CT compared with CBCT in lung biopsies: a retrospective cohort study
Source: Front Oncol. 2026 Mar 12;16:1758732. doi: 10.3389/fonc.2026.1758732 (PMC13019481; doi:10.3389/fonc.2026.1758732)
Supplement: Supplementary file 1 [file DataSheet1.docx]

Supplementary Material

# Supplementary Figures


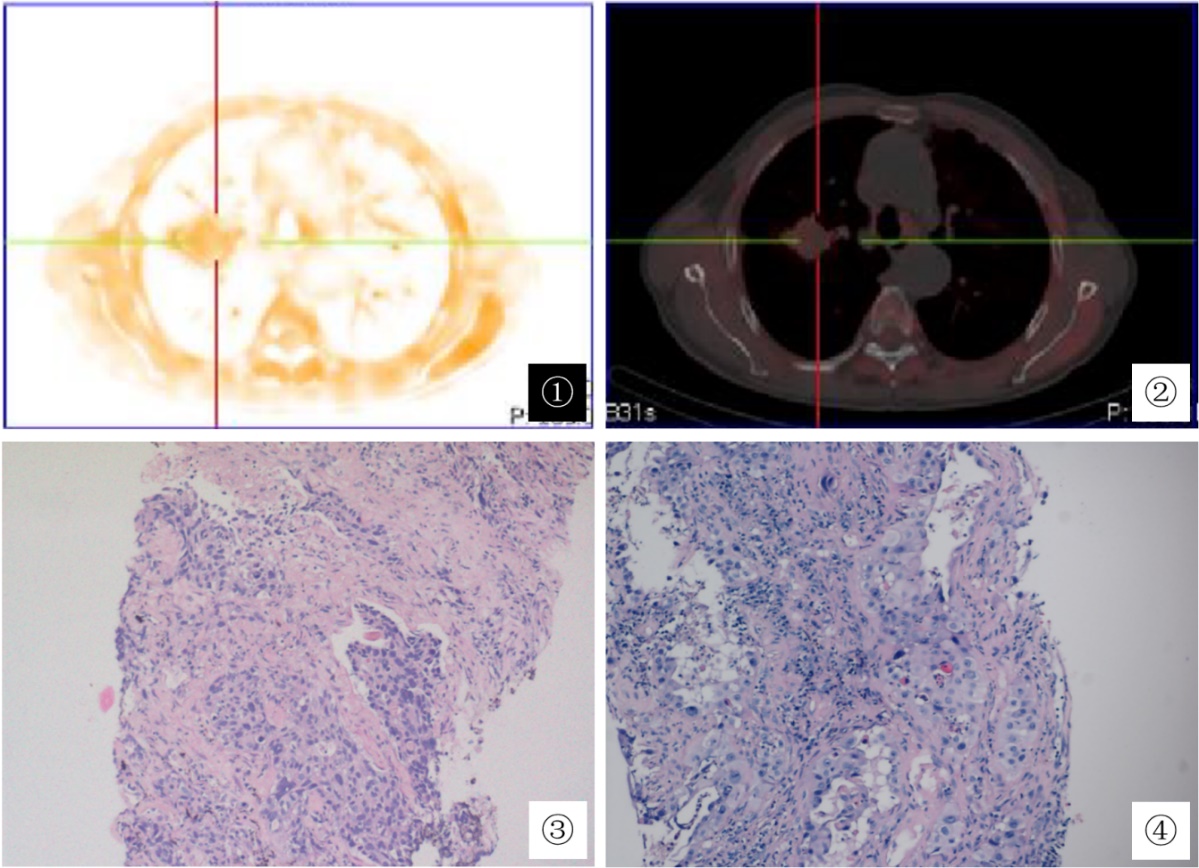


**Supplementary Figure 1.** **Case 1: Solitary nodule type.**

Note: A 72-year-old male with isolated nodule (2.8 cm) in the upper lobe of the right lung. ①^99m^Tc-MIBI SPECT imaging ②^99m^Tc-MIBI SPECT/CT tomography fusion imaging: radiological inhomogeneous concentration, mainly distributed in the lateral margin of the nodule. ③Puncture biopsy pathology: microscopic suggestion of adenocarcinoma, immunohistochemistry results: CK, TTF-1 are (+), CD50, NapsinA, P63, P40 are (-), Ki-67 positivity rate of about 80%. ④Surgical resection was performed 1 week later, and the pathologic results of the surgical resection specimen showed adenocarcinoma, which was consistent with the pathologic results of the preoperative puncture biopsy.


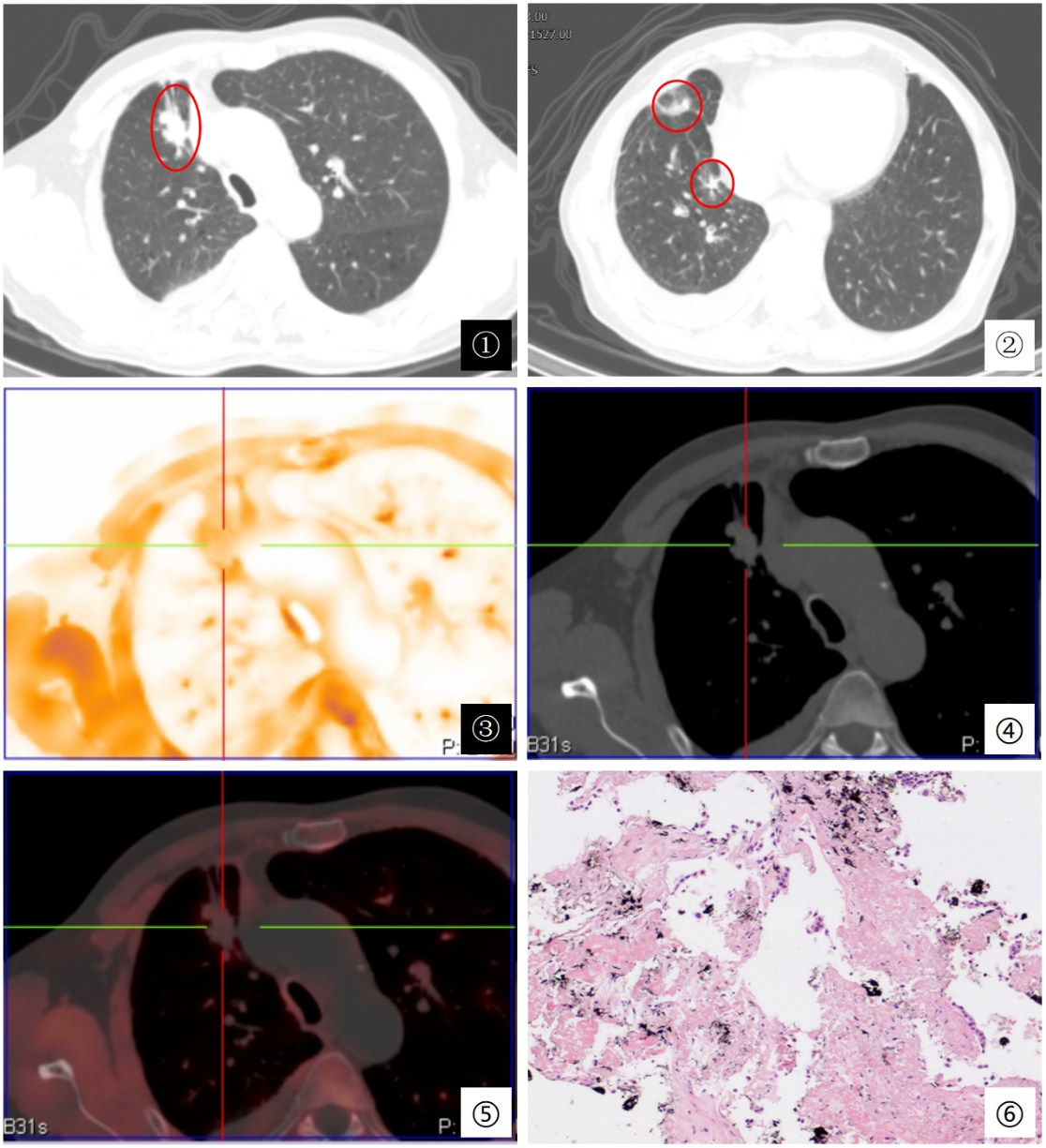


**Supplementary Figure 2. Case2: Multinodular nodule type**

Note: A 68-year-old male with multiple nodules in both lungs. ① Nodule in the upper lobe of the right lung (circled in red) ② Small nodule in the lower lobe of the right lung (circled in red). ③^99m^Tc-MIBI SPECT imaging ④CT tomography imaging ⑤^99m^Tc-MIBI SPECT/CT tomography fusion imaging: radiologically inhomogeneous concentration of nodules in the upper lobe of the right lung, mainly in the anterolateral margin of the nodule. ⑥ Puncture biopsy pathology: microscopic suggestion of alveolar epithelial heteromorphic hyperplasia with obvious interstitial fibrosis, consistent with lung adenocarcinoma. The patient was diagnosed with lung adenocarcinoma 1 year ago, and on this admission, multiple nodules were found in both lungs. In order to evaluate the condition, percutaneous lung puncture biopsy was performed, and the result was confirmed to be intrapulmonary metastasis of lung adenocarcinoma.

s
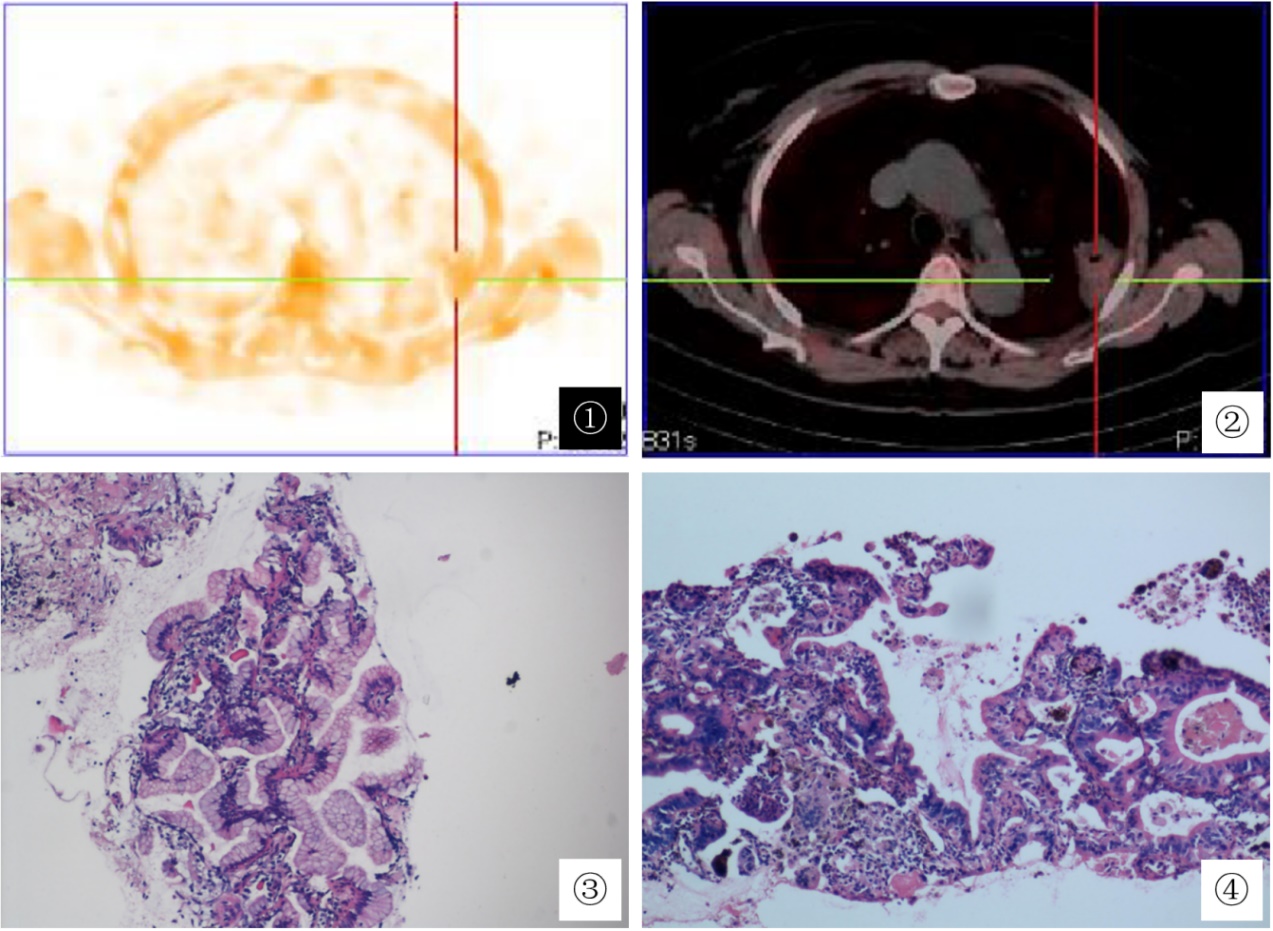


**Supplementary Figure 3. Case 3:Cavitary type**

Note: A 65-year-old female with a cavity (3.7 cm) in the lower lobe of the left lung. ①^99m^Tc-MIBI SPECT imaging ②^99m^Tc-MIBI SPECT/CT tomography fusion imaging: inhomogeneous concentration of radioactivity, mainly at the posterior edge of the cavity. ③Puncture biopsy pathology: microscopic suggestion of adenocarcinoma. ④ Surgical resection was performed 10 days later, and the pathologic results of the surgical resection specimen showed adenocarcinoma, which was consistent with the preoperative puncture biopsy pathologic results.


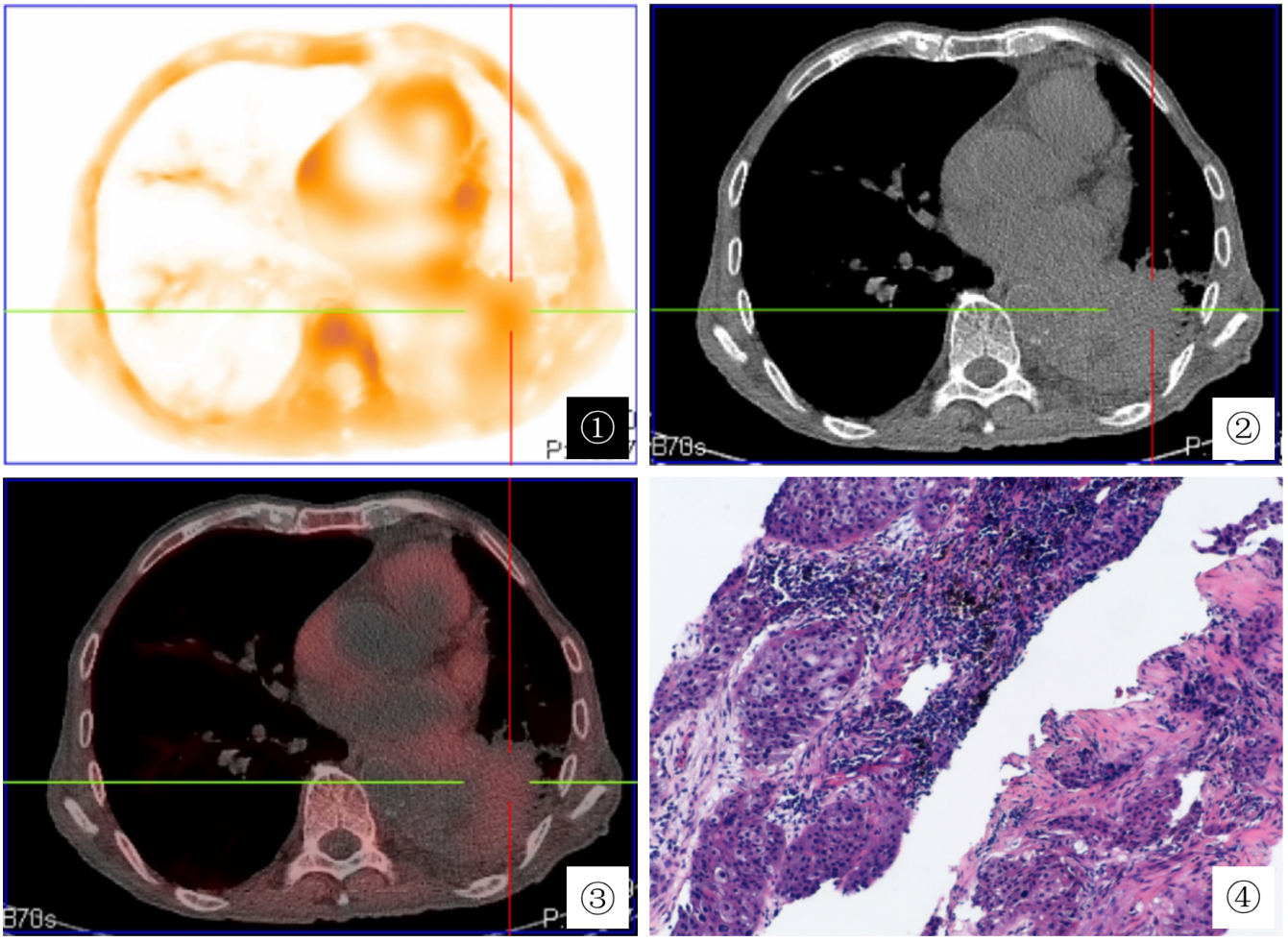
\

**Supplementary Figure 4. Case 4: Mass type (peripheral)**

Note: A 78-year-old male with mass type in the lower lobe of the left lung. ①^99m^Tc-MIBI SPECT imaging ②CT tomography ③^99m^Tc-MIBI SPECT/CT tomography fusion imaging: radiological inhomogeneous concentration, mainly distributed in the lateral margin of the lesion. ④ Puncture biopsy pathology: cancer was detected microscopically, immunohistochemistry was positive for CK, P63 and P40, and negative for TTF-1 and NapsinA. TTF-1 and NapsinA were all negative. It was suggested to be squamous cell carcinoma. The patient did not undergo surgery and was treated with chemotherapy in the oncology department of our hospital.


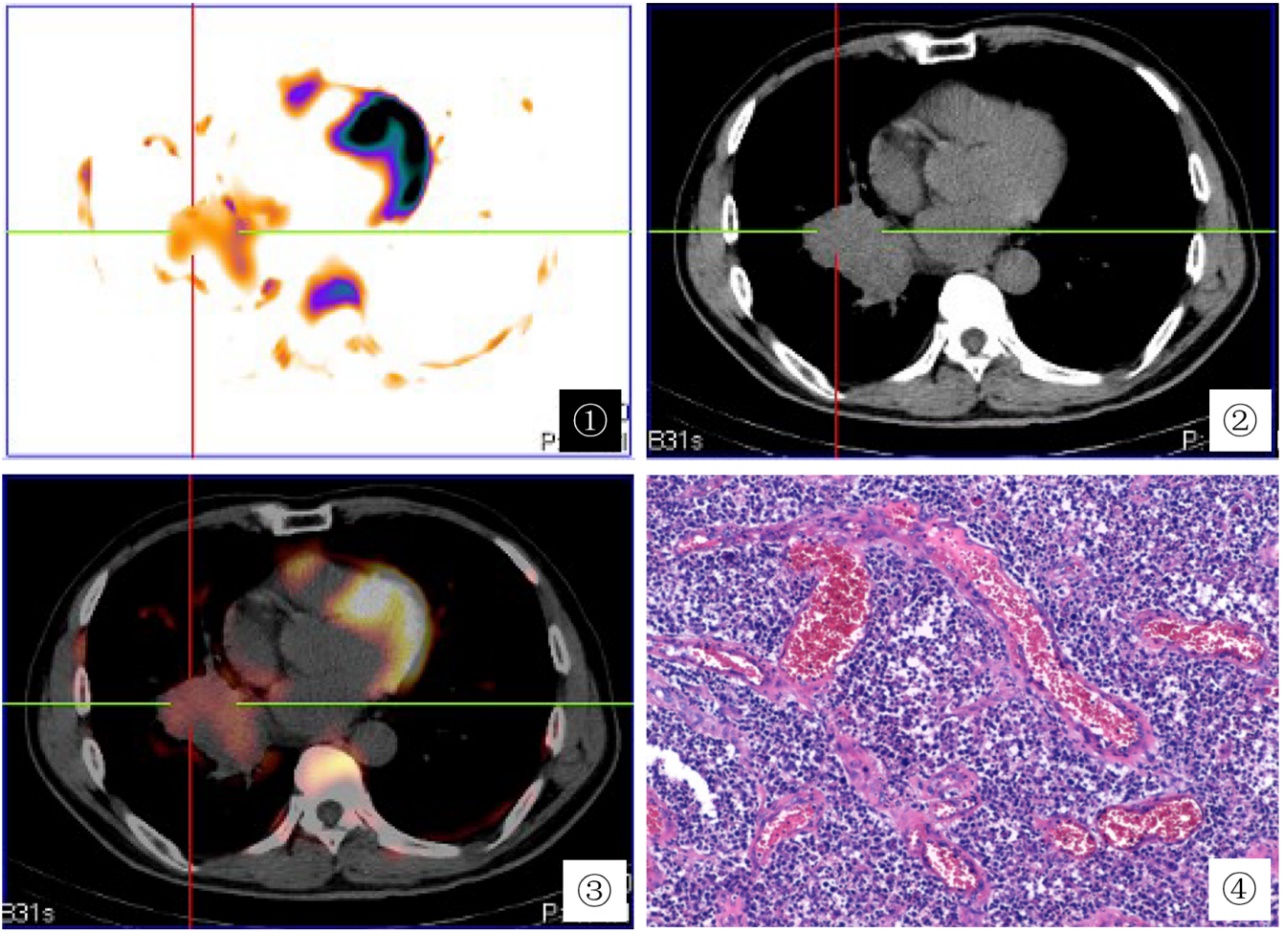


**Supplementary Figure 4. Case 5: Mass type (hilum)**

Note: A 57-year-old male with right hilar occupancy. ①^99m^Tc-MIBI SPECT imaging ②CT tomography ③^99m^Tc-MIBI SPECT/CT tomography fusion imaging: radiological inhomogeneous concentration, mainly in the medial margin of the lesion and mediastinal lymph nodes. ④Puncture biopsy pathology of the right hilar mass: microscopic examination of cells of large size, immunohistochemistry of PCK (-), CD10 (-), NapsinA (-), CD20 (-), CD3 (+), TTF-1 (-), Ki67 (+, about 30%), suspected of lymphatic system tumors, pathological specimens sent to the higher level hospitals for examination, and the results suggested that the non-Hodgkin's lymphoma, aggressive T lymphoma. He was treated with chemotherapy in the Department of Hematology of our hospital.
